# Supplementary material for: Scaling of a Large-Scale Simulation of Synchronous Slow-Wave and Asynchronous Awake-Like Activity of a Cortical Model With Long-Range Interconnections
Source: Front Syst Neurosci. 2019 Jul 23;13:33. doi: 10.3389/fnsys.2019.00033 (PMC6664086; doi:10.3389/fnsys.2019.00033)
Supplement: Supplementary file 1 [file Data_Sheet_1.PDF]

Supplementary Material - Scaling of a large-scale simulation of synchronous slow-wave and asynchronous awake-like activity of a cortical model with long-range interconnections

1 INITIAL CONSTRUCTION OF CONNECTIVITY INFRASTRUCTURE

During the initialization phase, each process contributes to an awareness about the subset of processes that should be listened to during subsequent simulation iterations. At the end of this construction phase, each "target" process should know about the subset of "source" processes that need to communicate with it, and should have created its database of locally incoming axons and synapses. A simple implementation of the construction phase can be realized using two steps. During the first step, each source process informs other processes about the existence of incoming axons and about the number of incoming synapses to be established. A single word, the synapse counter, is communicated among pairs of processes. Under MPI, this can be achieved by an MPI_Alltoall(). Performed once, and with a single-word payload, the cost of this first step creates a cumulative network load proportional to the square of the number of processes. The cost of this operation is negligible in the range of processes explored by this paper. The second step transfers the identities of synapses to be created on each target process. Under MPI, the payload, a list of synapses specific for each pair in the subset of processes to be connected, can be transferred using a call to the MPI_alltoallv() library function. The cumulative load created by this second step is proportional to the product of the total number of processes and the subset of target processes reached by each source process. The first step produces two effects: (1) it reduces the cost of the initial construction of synapses, the second step of the construction phase; and (2) the knowledge about the non-existence of a connection between a pair of processes can be used to reduce the cost of spiking transmission during the simulation iterations.

2 DELIVERY OF SPIKING MESSAGES DURING THE SIMULATION PHASE

Here, we describe the present implementation of the delivery of spiking messages. In this first implementation, we did not take advantage of the possibility of delivering spikes to targets just before the deadline imposed by the synaptic-specific delay. Instead, we used a synchronous approach: all spikes are delivered to target processes before proceeding to the simulation of the next time iteration of the neural dynamic. The delivery of spiking messages can be split into two steps, with communications directed toward subsets of decreasing sizes. During the first step, single-word messages (spike counters) are sent to the subset of potentially connected target processes. On each pair of source-target process subsets, the individual spike counter gives information about the actual payload (i.e., axonal spikes) that will have to be delivered, or about the absence of spikes to be transmitted between the pair. The knowledge of the subset has been created during the first step of the initialization phase, described in a previous section. The second step uses the spiking counter information to establish a communication channel solely between pairs of processes that actually need to transfer an axonal spike payload during the current simulation time iteration. In MPI, both steps can be implemented using calls to the MPI_Alltoallv() library function. However the two calls establish actual channels among sets of processes of decreasing size, as described previously.

3 SIMULATION PARAMETERS

The connectivity of a single module can be fully described by setting the values of recurrent synaptic efficacies (J^0_{ts}) and of external stimuli ($J^0_{t,ext}$). According to mean-field theory, specific values must be set depending on the network state that has to be simulated. Table S1 summarizes the values of synaptic efficacies, both for recurrent and external connectivity and for each simulated state.

The dynamic of the LIF neurons with SFA, used throughout all the simulations reported in this paper, is described by equation 2 in the main article. The values of all the parameters are summarized in Table S1.

Table S1. Simulation parameters. A summary of recurrent synaptic efficacies, external stimulus, and neural dynamics parameters. Recurrent synapses: $J_{t,s}$ are the values of synapses connecting the target neuron *t* with the source neuron *s*, for each simulated state. External stimulus: $J_{t,ext}$ represents the mean value of the efficacy of external synapses afferent the target neuron *t*; $\nu_{t,ext}$ represents its mean firing rate; $N_{t,ext}$ is the number of external Poissonian trains per target neuron *t* for each simulated state. Neural dynamics parameters: excitatory and inhibitory neurons are modeled according to equation 2 in the main article. Inhibitory neurons have no adaptation, therefore the second equation does not apply.

Recurrent synapses									
State	$J_{F,F}$	$J_{B,F}$	$J_{I,F}$	$J_{F,B}$	$J_{B,B}$	$J_{I,B}$	$J_{F,I}$	$J_{B,I}$	$J_{I,I}$
SW 3.1 Hz	0.600	0.382	0.560	0.382	0.429	0.560	3.17	3.17	3.0
AW 2.8 Hz	0.515	0.412	0.560	0.412	0.429	0.560	-1.5	-1.5	-1.5
AW 8.8 Hz	0.515	0.412	0.560	0.412	0.429	0.560	-1.5	-1.5	-1.5

External stimulus									
State	$J_{F,ext}$	$J_{B,ext}$	$J_{I,ext}$	$\nu_{F,ext}$	$\nu_{B,ext}$	$\nu_{I,ext}$	$N_{F,ext}$	$N_{B,ext}$	$N_{I,ext}$
SW 3.1 Hz	0.832	0.858	1.120	3.17	3.17	3.0	400	400	400
AW 2.8 Hz	0.858	0.858	1.120	3.17	3.17	3.0	400	400	400
AW 8.8 Hz	1.416	1.416	1.120	3.17	3.17	3.0	400	400	400

Neural dynamics parameters									
Neural kind	τ_m (ms)	C_m (pF)	E (mV)	V_θ (mV)	V_r (mV)	τ_{arp} (ms)	α_w (mV)	τ_w (ms)	g_w (nS)
Exc	20	1	0	20	15	2	1	1000	0.02
Inh	10	1	0	20	15	1	-	-	-
